# What Is It? A Rare Presentation of a Meningioma

**Published:** 2016-12-29

**Authors:** Matthew A. Applebaum, Connor Barnes, Michael Harrington

**Affiliations:** ^a^University of South Florida Morsani College of Medicine, Tampa; ^b^Division of Plastic Surgery, Department of Surgery, University of South Florida, Tampa

**Keywords:** meningioma, reconstruction, orbit, extracranial, temporal

## Abstract

**Introduction:** Primary extracranial meningiomas are rare manifestations of a central nervous system tumor. This article presents a case study of a soft-tissue primary extracranial tumor in the temporal region that was initially diagnosed as melanoma at an outside institution and whose definitive diagnosis proved difficult prior to successful excision. **Methods:** Temporal muscle biopsy, ultrasound-guided biopsy, and computed tomography were conducted at an outside institution prior to the patient's presentation to our care. Upon presenting to our institution a positron emission tomographic scan was then conducted prior to excision. After excision, the mass was sent to pathology and further immunohistochemistry was conducted. To ensure the mass was completely excised, magnetic resonance imaging was performed after its removal. **Results:** A 3 × 3-cm mass was excised in its entirety from the patient's temporal region and sent to pathology for immunohistochemistry and mutation testing. It proved to have the most common mutation for a primary extracranial meningioma, a neurofibromatosis type 2 frameshift. **Conclusion:** The presentation of a primary extracranial meningioma in the temporal region is a rare finding. Because of its slow-growing nature and generally asymptomatic presentation, it can be misdiagnosed. Utilization of radiological imaging is essential both pre- and postoperatively in order for its identification and complete excision.

Meningiomas are the most common primary brain tumor and account for approximately 18% of diagnosed intracranial tumors.^1^^,^^2^ Comparatively, the incidence of primary extracranial meningiomas is 1% to 2%.^1^^,^^3^ A relatively benign tumor, a primary extracranial meningioma has a variable presentation and is often misdiagnosed because it is rare, has nonspecific presentation, and has a slow-growing nature.

We present a case report of an 82-year-old woman with an asymptomatic swelling in the right temporal region that was present for 1 year. It measured approximately 5 × 4 cm and was not associated with any cutaneous changes. There was no history of trauma, radiation, infection, or genetic abnormality.

Before presenting to our institution, the patient had visited with multiple outside specialists in the fields of primary care, neurology, and otolaryngology. These outside facilities conducted an extensive workup that included a computed tomographic (CT) scan, a temporal muscle biopsy, and finally an ultrasound-guided biopsy of the mass. CT findings showed a mass lateral to the lateral orbital wall that was deep to the temporalis muscle. The pathology of the muscle biopsy from the outside hospital was positive for S100, vimentin, and HMB45 markers. These results increased the outside facilities suspicion for a malignant melanoma. Histological sections from the ultrasound-guided biopsy showed fragments of poorly differentiated tumor cells within the fibroadipose tissue and skeletal muscle with an epithelioid morphology. After the patient had these studies performed at the outside facility, she presented to our institution for a second opinion to aid in the diagnosis of the mass.

## METHODS

**Figure 1:**
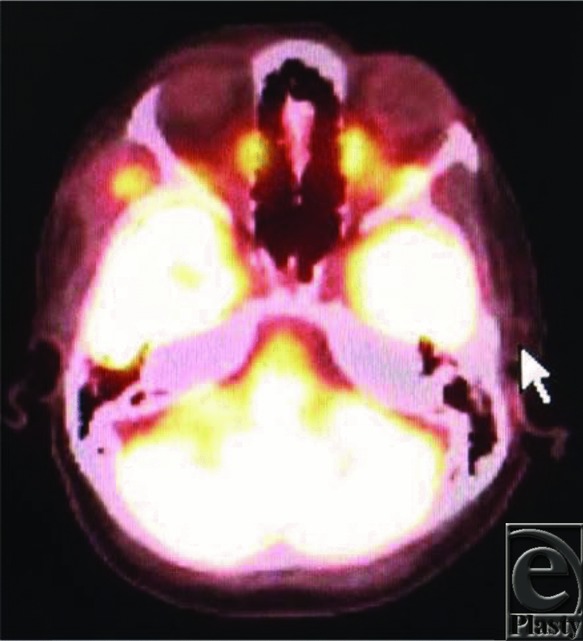
Positron emission tomography scan.

On presentation to our institution, a positron emission tomographic scan was ordered to look for possible malignancies. It showed a hypermetabolic soft-tissue swelling along the right temple with no other metastatic or primary lesions (Fig 1). The decision was made to perform an excision of the mass under general anesthesia and to examine it with immunohistochemistry (IHC). Excision of the mass was performed through the previous temporal muscle biopsy incision site because of the location noted on the CT scan from the outside institution. The mass was sent to pathology, where upon IHC was performed to help in its identification. Magnetic resonance imaging (MRI) was then conducted at the conclusion of the procedure to ensure that the mass was removed in its entirety.

## RESULTS

Prior to excision, the patient was noted to have paresis of the muscles supplied by the temporal branch of the facial nerve. This may have been secondary to the previous temporal muscle excision, during the first surgical procedure.

**Figure 2:**
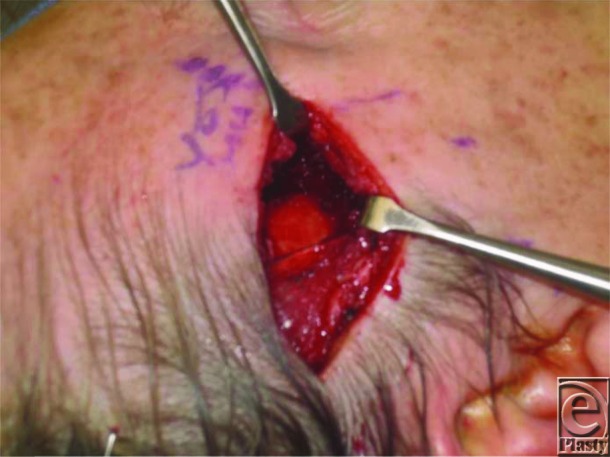
Excision of meningioma.

**Figure 3:**
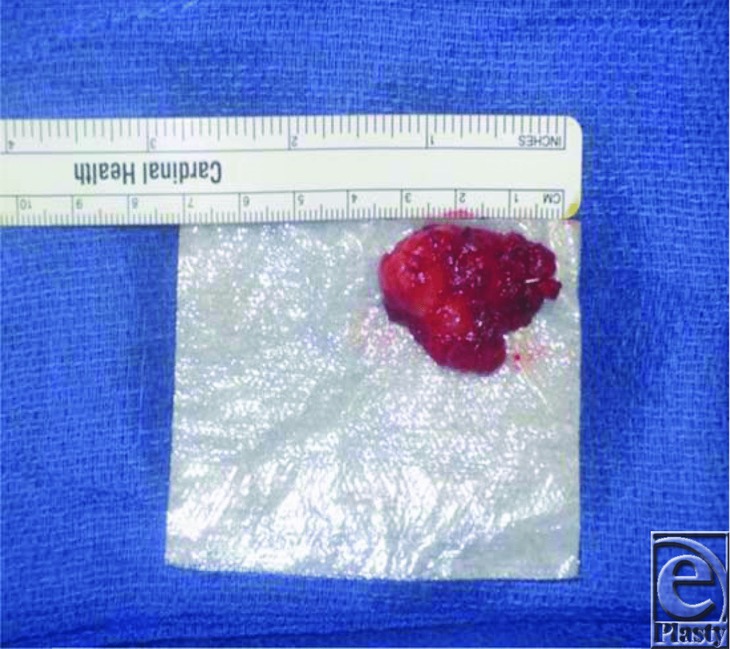
Excised meningioma measuring 3 × 3 cm.

Upon excision, the mass was measured to be 3 × 3 cm and was sent for a frozen section procedure to determine the type of mass (Figs 2 and 3). It had no osseous adhesions and showed no signs of erosion into the bone. The biopsy showed cytologically bland neoplastic cells and syncytial lobules with distinct cell borders, growing with whorled/nodular architecture as well as infiltrating the surrounding skeletal muscle. IHC was positive for p63 and CD31 but negative for melanoma markers S100, SOX-10, AE1/AE3, CK5/6, CK7, CAM, Actin MS, calponin, CD34, and CK8/18 and pan melanoma. Additional stains were positive for vimentin, epithelioid membrane antigen, and progesterone receptor. These findings, in addition to the clinical presentation, were suggestive for a possible meningioma. In view of the rare nature of this tumor, we further characterized it by conducting mutation testing with the FoundationOne panel. A neurofibromatosis type 2 (NF2) frameshift mutation was found, which is the most commonly mutated gene in meningiomas. AXIN1, CTNNB1, and PRKC1 mutations were also seen but were of unclear significance.

Postoperatively, MRI was conducted to confirm the complete excision of the mass and showed findings consistent with gross total tumor resection with expected postsurgical changes. We did not find intra-axial brain masses or enhancement throughout the brain or meninges. An incidental empty sella was found.

## DISCUSSION

Meningiomas are slow-growing benign tumors that usually originate from arachnoid cells and reside in the dura. With a 2:1 female predominance, as well as a mean age of diagnosis of 51.4 years, they have a predilection to grow intracranially.^4^^-^6 Almost 20% of extracranial manifestations are due to extension of a primary intracranial meningioma. Comparatively, primary extracranial meningiomas make up only 1% to 2% of all meningiomas and are most commonly seen in the head and neck.^1^^,^^3^^,^^7^ One study examined 146 cases of primary extracranial meningiomas and found that they predominantly originated from the skin and the scalp, followed by the middle ear and the sinonasal tract, with other rare cases being seen in the temporal region and the orbit.^1^^,^^8^ In addition to its rarity, the presentation within the temporal region, as in our patient, tends to present at a later age when compared with other primary extracranial meningiomas (50.1 compared with 36.2 years of age, respectively).^8^

Gross examination can be variable, ranging from gray to pink in color with a firm to rubbery consistency.^8^^,^^9^ There is also variability in IHC, leading to the formation of 4 different subtypes. Meningothelial is the most common type seen in soft-tissue manifestations (42.9%), followed by the psammomatous subtype (14.3%), with anaplastic and transitional being the least common (7.1% and 7.1%, respectively).^8^

The symptom of extracranial meningiomas is variable due to its possibility for different anatomical locations. It often can present with nonspecific signs that may lead practitioners to manage a patient's symptoms without a proper diagnosis. It is not uncommon for a patient to go up to 10 years without a diagnosis of extracranial meningioma.^1^ This may, in part, be due to how they are theoretically believed to manifest.

There are 4 theoretical explanations for how primary extracranial meningiomas manifest as proposed by Rushing et al^8^: (1) arachnoid cells are in fact present within nerve or vessel sheaths, allowing them to emerge from the skull; (2) throughout embryological development, pacchionian bodies are deposited extracranially; (3) the displacement of arachnoid cells may be due to trauma or cerebral hypertension; and (4) undifferentiated cells.^8^^,^^9^ There may also be possible associations with radiation, viral infection, NF2, or abnormalities in estrogen, progesterone, or androgens.^5^ We sought to find possible causes to our patient's manifestation.

Upon further examination, an NF2 frameshift mutation was unveiled in our patient, which had been previously undiagnosed. This likely caused an abnormal growth pattern of arachnoid cells, leading to the development of the extracranial meningioma. Furthermore, the initial misdiagnosis may have been due to an inadequate biopsy or a reliance on the CT scan that has not proven to be effective on its own in the diagnosis of an extracranial meningioma.

## CONCLUSION

This case report consisted of a rare manifestation of a primary extracranial meningioma. Preoperative and postoperative scanning with CT or MRI is vital to ensure that the entire mass has been or will be excised. This is because regrowth from incomplete excision is more common compared with recurrence.^9^ There was no intracranial component present on imaging in our patient and with the assistance of IHC possible confounders in the differential diagnosis such as osteoma, lipoma, or plasmacytoma were limited.^4^ These studies showed a nontypeable, soft-tissue mass on the CT scan and a whorled pattern on IHC that was consistent with the diagnosis of meningioma. Using the gold standard, the mass was excised in its entirety with negative margins.^8^^,^^9^ There has been no evidence of recurrence, and the patient has since recovered.
